# Supplementary material for: Prevalence of Orthosomnia in a General Population Sample: A Cross-Sectional Study
Source: Brain Sci. 2024 Nov 6;14(11):1123. doi: 10.3390/brainsci14111123 (PMC11592250; doi:10.3390/brainsci14111123)
Supplement: Supplementary file 1 [file brainsci-14-01123-s001.zip › brainsci-3256543-supplementary.pdf]

Supplementary Materials

```
# Assuming your data frame is called 'data' with columns variables as [GAD7, Wearable, AIS, APSQ]

# Create a new column 'orthosomnia' in the data frame

data$orthosomnia <- with (data, ifelse(GAD7 <= 14 & Wearable== Yes & AIS >= 6 & APSQ >= 40,
"Case", "Noncase"))

# Calculate the frequency and percentage of orthosomnia cases

ortho_freq <- table(data$orthosomnia)

ortho_percent <- prop.table(ortho_freq) * 100

# Create a data frame with the results

results <- data.frame(Orthosomnia = names(ortho_freq), Counts = as.vector(ortho_freq),
Percentage = as.vector(ortho_percent), Cumulative_Percentage =
cumsum(as.vector(ortho_percent)))

# Print the results

print(results)

# Quick summary

summary(data$orthosomnia)
```

The complete code is available at https://github.com/hjahrami/
